# Event-related potentials as neurophysiological predictors of high-risk isolated REM sleep behavior disorder patients

**DOI:** 10.1093/sleep/zsae311

**Published:** 2025-01-01

**Authors:** Giuseppe Lanza, Maria P Mogavero, Raffaele Ferri

**Affiliations:** Clinical Neurophysiology Research Unit and Sleep Research Centre, Oasi Research Institute-IRCCS, Troina, Italy; Department of Surgery and Medical-Surgical Specialties, University of Catania, Catania, Italy; Vita-Salute San Raffaele University, Milan, Italy; Sleep Disorders Center, Division of Neuroscience, San Raffaele Scientific Institute, Milan, Italy; Clinical Neurophysiology Research Unit and Sleep Research Centre, Oasi Research Institute-IRCCS, Troina, Italy

The identification of early biomarkers for neurodegenerative diseases remains a pivotal challenge in medical research. Isolated rapid eye movement (REM) sleep behavior disorder (iRBD) is increasingly recognized as a prodromal stage of alpha-synucleinopathies, such as Parkinson’s disease (PD), dementia with Lewy bodies (DLB), and multiple system atrophy [1]. The clinical importance of this recognition lies in its potential to provide a window of opportunity for early intervention, possibly altering the trajectory of these disabling conditions [2]. However, predicting which patients with iRBD will progress to overt neurodegenerative diseases—a process known as phenoconversion—has proven to be highly complex [1]. In this framework, the study by Choi et al. [3] makes a significant contribution to this field by demonstrating that event-related potentials (ERPs) elicited during visuospatial attention tasks could serve as a reliable predictor of phenoconversion in iRBD patients.

At the heart of this study is the investigation of specific neurophysiological changes associated with attention processes in iRBD patients. Using the Posner visuospatial cueing paradigm, the researchers examined ERP components elicited by cue and target stimuli. These components, specifically N2 and P3, were analyzed for their amplitude and timing, providing insights into the neural mechanisms underlying attentional orientation and cognitive control. To this end, the study enrolled 126 iRBD patients and followed them longitudinally during an average follow-up period of 6.3 years to determine phenoconversion status, classifying participants into converters (iRBD-CV) and non-converters (iRBD-NC). A group of healthy controls was also included to establish baseline comparisons. The findings reveal that iRBD patients, as a whole, exhibited significant reductions in N2 amplitudes across various conditions compared to healthy controls. This reduction points to impairments in early attentional processes, which are critical for efficient visuospatial functioning. More importantly, when comparing iRBD-CV and iRBD-NC groups, distinct patterns emerged in cue-elicited ERPs. Namely, the iRBD-CV group displayed a pronounced increase in P3 amplitude and a decreasing trend in N2 amplitude in response to cue stimuli. These alterations were strongly associated with faster rates of phenoconversion. The study’s survival analysis further underscored the predictive value of these biomarkers, showing that reduced N2 and enhanced P3 amplitudes correlated with shorter times to phenoconversion.

Overall, these results provide compelling evidence that ERPs elicited during attentional tasks may reflect underlying neurodegenerative processes even before the onset of clinical symptoms. The observed increase in P3 amplitude among converters may represent a compensatory mechanism, wherein the brain attempts to mobilize additional cognitive resources—the so-called “cognitive reserve” [4]—to maintain function despite progressive neurodegeneration. This hypothesis aligns with existing literature suggesting that compensatory hyperactivation often precedes overt cognitive decline in neurodegenerative disorders [5, 6]. Conversely, the reduction in N2 amplitude indicates deficits in the preparatory and orientation phases of attention, suggesting that these impairments are among the earliest manifestations of alpha-synucleinopathy-related neurodegeneration.

These findings align with and further reinforce previous evidence that some neurophysiological measures are strong predictors of, or at least correlate with, neurodegenerative processes in iRBD [7]. For instance, studies by Ferri et al. have demonstrated that the REM Atonia Index [8, 9], derived from electromyographic activity during REM sleep, is a sensitive marker of motor system dysfunction and an early indicator of neurodegeneration in iRBD patients [10, 11]. Similarly, research by Puligheddu et al. has highlighted the predictive value of vestibular evoked myogenic potentials, which assess brainstem integrity and have been shown to correlate with the risk of phenoconversion in RBD [12, 13]. Moreover, studies by Lanza et al. have demonstrated the utility of motor-evoked potentials to transcranial magnetic stimulation in detecting early cortical dysfunction in RBD, offering further evidence of neurophysiological measures as effective biomarkers for impending neurodegeneration [14, 15]. The current study by Choi et al. [3] complements these findings by focusing on cognitive and attentional pathways, showcasing the broad potential of neurophysiological tools in identifying individuals at high risk for alpha-synucleinopathies.

The clinical implications of these findings are relevant. By establishing ERP-based biomarkers as predictors of phenoconversion, this study opens the door to more targeted monitoring and stratification of iRBD patients. Patients identified as high-risk based on their ERP profiles could be prioritized for closer follow-up, more aggressive risk mitigation strategies, or even enrollment in clinical trials for neuroprotective therapies. Moreover, these biomarkers may facilitate earlier diagnosis of alpha-synucleinopathies in their preclinical stages, enabling interventions aimed at delaying or preventing the onset of motor and cognitive symptoms.

Despite its strengths, the study does have some limitations that warrant discussion. The relatively small number of converters limits the statistical power to detect subgroup-specific differences, such as those between patients progressing to PD versus DLB. Furthermore, the study’s reliance on cue-elicited ERPs leaves room for additional exploration of target-elicited components and their potential predictive value. Future research may also benefit from incorporating multimodal approaches, for example, combining ERP data with other biomarkers such as advanced imaging, genetic analyses, or measures of autonomic dysfunction [1]. This integrative approach will provide a more comprehensive picture of the diverse neurodegenerative pathways in iRBD. Expanding the scope of ERP analysis is another promising avenue for future investigation. While this study focused on long cue-to-target intervals to isolate mechanism of the impairment of inhibition of return, including shorter intervals could enhance our understanding of attentional dynamics and their disruption in iRBD. Advanced analytical techniques, such as machine learning, could also be employed to develop individualized risk models based on ERP and other neurophysiological data. Additionally, longitudinal studies with extended follow-up periods are needed to validate these findings and explore how ERP profiles evolve as patients’ approach phenoconversion.

The broader implications of this research extend beyond iRBD. By establishing a framework for using neurophysiological markers to predict disease progression, the study contributes to a growing body of work that seeks to bridge the gap between preclinical detection and clinical intervention in neurodegenerative diseases. This paradigm that shifts from reactive to proactive care has the potential to transform outcomes for patients and reduce the social burden of these conditions. As the field moves forward, integrating findings from studies like this into routine clinical practice will be a critical step in realizing the promise of personalized Medicine in general Neurology, and in sleep and movement disorders in particular.

In conclusion, this study underscores the potential of ERPs as a noninvasive and cost-effective tool for identifying iRBD patients at high risk of phenoconversion. The distinct alterations in cue-elicited N2 and P3 components not only provide valuable insights into the pathophysiology of alpha-synucleinopathies, but also offer a practical approach to early risk stratification. By enabling timely interventions, these biomarkers represent a significant advancement in the fight against neurodegenerative diseases. As research continues to refine and expand upon these findings, the hope of altering the course of diseases such as PD and DLB becomes increasingly tangible.
